# Cysteine proteases and how YabG fits into clan CD of the MEROPS database

**DOI:** 10.1128/jb.00246-25

**Published:** 2025-08-20

**Authors:** Morgan S. Osborne, Joseph A. Sorg

**Affiliations:** 1 Department of Biology, Texas A&M University 14736, College Station, Texas, USA; 2 Division of Infectious Diseases, Vanderbilt University Medical Center 192785 , Nashville, Tennessee, USA; The Ohio State University, Columbus, Ohio, USA

**Keywords:** spore, protease, *Bacillus subtilis*, *Clostridioides difficile*, MEROPS

## Abstract

Cysteine proteases are hydrolases that share a common catalytic mechanism involving a nucleophilic cysteine thiol in a catalytic dyad or triad. Here, we review the current clans that make up the cysteine proteases in the MEROPS database as of March 2025. We also discuss cysteine proteases made by *C. difficile*, with a particular focus on recent analysis of the sporulation-specific protease, YabG, that supports its reclassification into clan CD of the MEROPS protease database. YabG is a highly conserved sporulation-specific protease that, until more recently, has been mostly studied in *B. subtilis,* where YabG is important for processing coat proteins. In *C. difficile*, YabG processes proteins required for spore germination and is important in coat/exosporium protein expression.

## INTRODUCTION

The MEROPS database is a database of peptidase classification, where proteases are grouped into one of 66 clans. After clan classification, the proteases remain unassigned or are further classified into one of 281 families (1). Proteases classified into families are defined as having an evolutionary relationship based on similar tertiary structure, primary sequence order of catalytic residues if the structure is not available, or common sequence motifs near the catalytic residues. Based on catalytic mechanisms, proteases are classified into six distinct classes: aspartic, glutamic, metallo, cysteine, serine, and threonine proteases.

In the MEROPS database, there are 1,071 cysteine proteases that remain unclassified or are classified into one of 11 clans, where C denotes it is a cysteine protease; CA, CD, CE, CF, CL, CM, CN, CO, CP, CQ, and CR (1). Among all the cysteine protease clans, there are 101 families. Some families are further divided into subfamilies due to evidence of divergence within the family.

Cysteine proteases are found in all domains of life, and many are synthesized as inactive precursors, or zymogens, where a propeptide or prodomain blocks their catalytic site and prevents unwanted protein degradation until the zymogen is activated. This prodomain can also have other functions, such as facilitating protein folding or intracellular trafficking of the peptide (2, 3). Activation of the zymogen requires removal of the prodomain, which can be accomplished by autocatalytic processing, trans-processing, or changes in pH (4, 5). Many cysteine proteases contain a Cys-His-Asn/Asp catalytic triad, while others contain a Cys-His catalytic dyad. For catalysis, the histidine residue serves as a proton donor to the cysteine. This allows the thiol of the active site cysteine to act as a nucleophile, which then attacks the carbon present in the carboxyl group next to the peptide bond in the target protein (4, 6, 7). Cysteine proteases that are in the same clan contain the same sequence motif of catalytic residues. Founding members of the clan are the basis for which other proteases are compared. As new evidence arises (such as crystal structures and predictive modeling), proteases can be moved to new families within the clans or new clans generated that are more representative of their characteristics.

## CLAN CA

Clan CA contains 46 families of cysteine proteases and is the largest of the cysteine clans (1). All of these enzymes have a Cys, His, Asn (or Asp) catalytic motif and have structural similarity to papain (8). Papain is a protease extracted from the papaya plant, *Carica papaya*, and was one of the first enzymes characterized and structure determined (8). Papain-like proteases contain a Cys and His catalytic dyad and two other important residues: the Gln that precedes the catalytic Cys and the Asn/Asp that follows the catalytic His residue. Papain has broad substrate specificity, although it has a preference for arginine at the P1 position (the amino acid immediately N-terminal to the scissile bond) and Val/Phe/Thr at the P2 position (the amino acid residue two positions upstream of the scissile bond) (9). Members of this clan mostly consist of endopeptidases, though there are several exopeptidases found in a variety of organisms. In *C. difficile,* the Cwp84 cysteine protease is a member of the clan CA and the C1 family (10–12).

## CLAN CD

The protease clan CD is comp0sed of eight families, where the families resemble the founding member, caspase-1. These include C11 (clostripain), C123 (RARP2), C13 (legumain), C14 (caspase-1), C25 (gingipain RgpA), C50 (separase), C80 (RTX self-cleaving toxin), and C84 (prtH peptidase) (1).

Members of clan CD are site-specific endopeptidases whose substrate specificity is driven by the identity of the P1 residue. Family C11 (clostripain) specifically cleaves after Arg residues; family C13 (legumain) specifically cleaves after Asp or Asn residue; family C14 (caspases) cleaves specifically after aspartate, although the metacaspases cleave after basic residues (Arg or Lys); and family 25 (gingipain) also cleaves after Arg or Lys (1, 13–20).

Caspase-1, a member of the C14 family, has a His-Cys catalytic dyad. This protease family specificity is dependent upon the specific caspase. For example, members of the subfamily C14A (caspases) have strict specificity for aspartyl bonds, whereas members of the C14B (metacaspases and paracaspases) subfamily have strict specificity for arginyl or lysyl bonds (21–23). Caspases are commonly held inactive as a zymogen and are activated upon apoptosis by cleavage on the carboxyl side of the specific aspartic acid residues, resulting in the alpha and beta subunits (24). Because many have functions in homeostasis, regulating inflammation, or cell death, their activation is tightly controlled (25, 26).

Clostripain was first identified in the endospore-forming *Hathewaya histolyticum* (formerly *Clostridium histolyticum* [27]), and a homolog is also found in *Clostridium perfringens* (28–30). Members of the C11 family have strict specificity for hydrolysis of arginyl bonds, with a conserved active site motif -His-Gly-(Xaa)_n_-Ala-Cys (31). Though clostripain is a major extracellular protease for *H. histolyticum*, it is non-essential for disease. The *C. perfringens* clostripain-like protease is reported to induce uptake of apoptotic neutrophils by macrophage phagocytosis (28). This protease is typically used in the laboratory as an effective arginine-specific peptidase for protein sequencing techniques like mass spectrophotometry and human islet isolation (32).

The RTX-self-cleaving toxin from *Vibrio cholerae* is a member of the C80 family that contains bacterial toxins that self-cleave via a cysteine peptidase mechanism and classified as a multifunctional autoprocessing repeats-in-toxin (MARTX) (33, 34). MARTX toxins are characterized by having glycine-rich repeat regions I the N- and C-termini and are secreted by the type I secretion system (T1SS), followed by a cysteine protease domain (CPD), where His and Cys make up the catalytic dyad (34–38). The glycine-rich repeats are hypothesized to form a pore within the host cell membrane (36, 39). The cysteine protease domain (CPD) is activated by the binding of host inositol hexakisphosphate (InsP_6_), resulting in autoprocessing after Leu2447, Leu3099, and Leu3441 releasing the Rho inactivation domain (RID) and α/β hydrolase (ABH) from the large holotoxin (34). The actin-crosslinking domain (ACD) and CPD remain attached to the N- and C-terminal membrane-bound regions (34, 39–43). The activation of ACD results in actin crosslinking leading to the depolarization of cells (44–46). Release of RID inactivates small Rho protein GTPases, blocking interaction with downstream effectors (47). In 2008, the tertiary structure of the *V. cholerae* RTX toxin was determined and, because of the structural similarities to caspase-1 (C14 family), was included in the clan CD (42).

Other members of this family are the *Clostridial* toxins TcdA and TcdB that are similar to MARTX family toxins. These large glucosylating toxins are the drive for the primary symptoms associated with *C. difficile* infection (CDI), severe diarrhea (48, 49). TcdA and TcdB contain a catalytic dyad composed of Asp, His, and Cys that are discussed more in depth later in this review (39, 50, 51).

## CLAN CE

Clan CE proteases are adenain-like proteases. Proteases in this clan are endopeptidases, and their catalysis is thought to be similar to papain (containing a His-Cys catalytic dyad). There are seven families in this clan: C5 (adenain), C122 (SdeA), C48 (Ulp1 peptidase), C55 (YopJ), C57 (vaccinia virus I7L processing peptidase), C63 (African swine fever virus processing peptidase), and C79 (ElaD peptidase) (1). Clan CE members are placed within this clan based upon similarity to adenain, an adenovirus endopeptidase, that is required for virion uncoating, maturation, and release from the infected cell (52–54). Members of this family possess active site residues in the order of His, Asp / Asn, Gln, and Cys (1).

## CLAN CF

Clan CF only has one family, C15, that contains the *Bacillus amyloliquefaciens* pyroglutamyl-peptidase I. This family contains omega peptidases (peptidases that cleave non-standard peptide bonds) that remove terminal residue that are substituted, cyclized, or linked to isopeptide bonds (55). Peptides carrying information, such as hormones, evade aminopeptidase degradation through the presence of an N-terminal pyroglutamyl (pGlu) residue (e.g.*,* the human hormone, thyrotropin-releasing hormone [TRH], which is produced in the hypothalamus and stimulates the pituitary gland) (56, 57). Members of the C15 family release an N-terminal pyroglutamate residue and have a catalytic triad of Glu, Cys, His (57).

## CLAN CL

Clan CL contains the C60 and C82 families, which contain sortase A and L,D-transpeptidase, respectively. Members of this clan are involved in bacterial cell wall hydrolysis and possess a His, Cys catalytic dyad. Sortases are transpeptidases found in the cell envelope of many Gram-positive bacteria, where they anchor surface proteins to the peptidoglycan cross bridge of the cell wall. Other sortases are involved in pilus assembly (58–60). L,D-transpeptidases catalyze the formation of 3-3 peptidoglycan cross-links in Gram-positive bacteria (61, 62).

## CLAN CM

Clan CM is composed of the C18 family, where the founding member is the hepatitis C virus peptidase 2 (also known as NS2/3). NS2/3 is a viral polyprotein endopeptidase and is an active homodimer. Each monomer contains catalytic residues His, Glu, and Cys. His and Glu from one monomer interact with Cys from the other monomer, and vice versa. The NS2/3 protease cleaves the HCV polyprotein between non-structural proteins NS2 and NS3, generating mature viral proteins (63, 64).

## CLAN CN

Clan CN only has one family, C9, that is made up of the Sindbis virus-type nsP2 peptidase. This peptidase has a Cys His catalytic dyad and is responsible for cleaving the non-structural proteins of the Sindbis virus (65). Originally believed to be a papain-like protease due to the similar catalytic dyad, the tertiary structure is unrelated and was thus the founding member of clan CN (66).

## CLAN CO

Clan CO is composed of the C40 family of dipeptidyl-peptidases. Members of this family are involved in cell-wall modification, e.g.*,* dipeptidyl-peptidase VI (DPP VI), a cytoplasmic peptidase of *Bacillus sphaericus*. During sporulation of *B. sphaericus*, DPP VI hydrolyzes γ-D-Glu-DAP(Lys) linkages in peptides that have a free N-terminal L-alanine (67). In the closely related *B. subtilis,* the cell wall lytic enzymes LytF, LytE, and CwlS are involved in cell wall separation (68–70). Members of this family have conserved cysteine and histidine residues that form a catalytic triad of Cys, His, His (71).

## CLAN CP

Clan CP is made up of the C97 family, deSUMOylating isopeptidase 1. SUMO (small ubiquitin-like modifier) deconjugating enzyme is a post-translational protein modification pathway in eukaryotes (72). The reversible attachment of SUMO is essential for correct activity, localization, and/or interactions with targets. Members possess a His, Cys catalytic dyad, the reverse of what is observed in papain-like proteases.

## CLAN CQ

The only member of CQ is the C53 family made up of pestivirus Npro peptidase. Family members are endopeptidases that process the *Pestivirus* p20 polyprotein and contain a His, Cys catalytic dyad (73). This autoprotease is located at the N-terminus of the polyprotein and releases the core protein using autolysis (74). Cleavage is observed between a Cys and Ser but can also follow Ala or Ser at the P1 site (74). The release of Npro blocks host interferon response by inducing degradation of interferon regulatory factor-3 (75, 76).

## CLAN CR

Clan CR is composed of the C108 family, the Prp peptidase identified in *Staphylococcus aureus*. Possessing a His, Cys catalytic dyad, this protease is important for processing the N-terminal extension of ribosomal protein L27 and is essential in *S. aureus* (77, 78). The *Streptococcus *phage CP-1 is also included in this family. CP-1 is an endopeptidase that cleaves the first 48 amino acids of the major head protein (79).

## *C. DIFFICILE* CYSTEINE PROTEASES

*Clostridioides difficile* is the causative agent of *C. difficile* infection (CDI) (80, 81). The severity of CDI can range from mild diarrhea to more severe pseudomembranous colitis and toxic megacolon (82). In 2019, the Centers for Disease Control and Prevention (CDC) estimated that there were 223,900 hospitalized cases and approximately 13,000 deaths caused by CDI (83). Moreover, the annual treatment-associated costs to the United States healthcare system are estimated to ~$5 billion (84, 85). Though CDI is a common occurrence in the healthcare setting, the incidence of community-acquired CDI has increased, particularly in immunocompromised patients (81, 84, 86, 87).

The infectious form of *C. difficile* is the spore because its vegetative cells are strictly anaerobic (88, 89). Spores are metabolically dormant, survive in the aerobic environment outside of the host, and permit host-to-host transmission (89–93). Upon ingestion, spores persist in the gut environment and germinate in response to host-derived bile acid germinants and amino acid co-germinants (88, 93–95).

As of the 2025 MEROPS database, many *C. difficile* strains encode approximately 192 known or putative proteases. One of the most well-studied is Cwp84, a member of clan CA. Cwp84 is a cell wall-localized cysteine protease and is responsible for processing the S-layer protein SlpA (96). The two *C. difficile* toxins TcdA and TcdB have a cysteine protease domain adjacent to the glucosyltransferase domain (GTD) and are members of clan CD (97). The loss of catalytic activity results in less cytotoxicity to the cell (50, 97–99).

The vegetative cells secrete the TcdA and TcdB toxins that are necessary and sufficient for disease symptoms (100–102). TcdA is a 308 kDa enterotoxin, and TcdB is a 270 kDa cytotoxin (100, 103, 104). Both toxins are classified as ABCD toxins, where there are four functional domains: A stands for biological activity; B, binding; C, cutting; and D, delivery (105). TcdA and TcdB are composed of a glucosyltransferase domain (GTD), a Clan CD cysteine protease domain, also known as the autoprocessing domain (APD), a delivery domain, and a receptor binding domain (50, 104, 106). The His and Cys catalytic residues of the TcdA/TcdB APD bind to Zn^2+^ and are essential for its autoprocessing activity (51, 107). These toxins bind to the target cell membrane through their C-terminal receptor binding domains, resulting in internalization of the toxin. After binding, the toxins are endocytosed to the endosomal compartment. A conformational change occurs that allows the toxin to interact with the endosomal membrane and generation of the pore. Autoprocessing occurs in the presence of host-derived InsP6 that binds to the adjacent domain of the monoglucosyltransferase that releases the N-terminal glucosyltransferase domain (GTD) into the host cytosol and targets Rho-family GTPases domain (50, 99, 105, 108, 109). The domain adjacent to the GTD of TcdA/TcdB shares sequence homology to MARTX toxins and is activated in the presence of InsP6 in the host cytosol (40, 110). GTPases are molecular switches that regulate cellular processes. Rho GTPases are involved in signaling processes, such as actin cytoskeleton regulation, phagocytosis, cell polarity, and more (111). The released GTD is then free to glucosylate Rho proteins, thus inactivating them. The result of this inactivation is cytopathic effects characterized by loss of actin stress fibers, disruption of intercellular tight junctions, increased cell barrier permeability, and eventually, cell death (100, 112–117). Interestingly, work done by Chumbler et al. (2012) revealed TcdB autoprocessing resulting in release of the GTD is not required for necrosis in epithelial cells (118). In addition to epithelial cell death, TcdA and TcdB cause inflammation in the epithelial cells induced by chemokines that promote neutrophil recruitment. The recruitment of neutrophils has been demonstrated to cause increased fluid secretion and enhanced mucosal inflammation, leading to diarrhea (119–122).

## YabG: AN UNCLASSIFIED CYSTEINE PROTEASE

YabG is a sporulation-specific cysteine protease found in all endospore-forming bacteria (123–127). In *C. difficile*, YabG processes two proteins that are critical for spore germination, CspBA and preproSleC, but is likely to have other unidentified targets in the *C. difficile* spore (127–130). Though much work has been done to study this protease, it remains unclassified in the MEROPS database (123–131).

In *B. subtilis*, YabG is responsible for proper processing of spore coat proteins. In *C. difficile*, YabG has been shown to both process proteins important for spore germination and regulate the incorporation of coat and exosporium proteins (123, 124, 126, 128–130). *B. subtilis* YabG has Cys218 and His172 catalytic residues required for autoprocessing (126).

**Fig 1:**
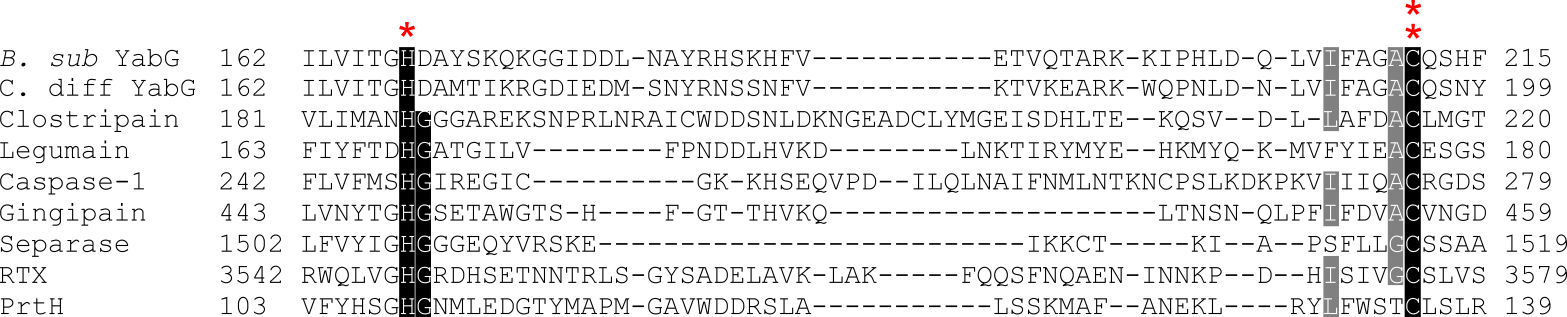
Sequence comparison of YabG to clan CD proteases. Alignment of catalytic residue amino acid sequences. *B. subtilis *168 YabG (*B. sub*) (P37548); *C. difficile* R20291 (*C. diff*) (A0A9R0BNR8); Clostripain from *H. histolytica* (P09870); Legumain from mouse (O89017); Caspase-1 from human (P29466); Gingipain from *P. gingivalis* (P28784); Separase from *S. cerevisiae* (Q03018); RTX from *V. cholerae* (Q9KS12); and PrtH from *T. forsythia* (O24742). The conserved His-Gly and Cys residues are highlighted in black. Residues highlighted in gray are 80% conserved. Red * indicates the YabG catalytic His, and red ** indicates the YabG catalytic Cys residues relative to other cysteine proteases.

Currently, YabG remains unclassified in the MEROPS database as U57. YabG possesses His-Cys catalytic residues with arginine specificity in the P1 position that are conserved across many endospore-forming bacteria (126, 129). In *C. difficile*, YabG has conserved His161, Asp162, and Cys207 residues that are required for autoprocessing (129). *B. subtilis* YabG possesses an Asp at position 173 that corresponds to *C. difficile* Asp162; however, the involvement in autoprocessing has not been tested (126). There are currently four cysteine protease clans that possess a His-Cys catalytic dyad: clans CD, CL, CP, and CQ (1). It was suggested by both Marini et al. and Yamazawa et al. that YabG be classified as a clan CD protease. Like other proteases within the clan CD, YabG does not share sequence homology with other members of this clan; however, the His-Cys catalytic dyad and Arg specificity make it a strong candidate for the CD clan (Fig. 1) (126, 128, 129). Members of the MEROPS database are not classified based on sequence similarity; rather, they are classified based on similar structure features and functions. The clan CD contains proteases with a highly conserved His-Cys or His-Cys-Asp/Asn catalytic dyad or triad. Other clans of cysteine proteases contain a His-Cys catalytic dyad, but they do not share the same function as those in clan CD. Members of the clan CD possess a Gly residue preceding the catalytic His residue. However, in YabG, the Gly residue following the His residue and the active Cys occurs in an Ala-Cys motif (129). This could result in a new family present within clan CD that contains enzymes with the same features as YabG.

## CONCLUDING REMARKS

Here, we present the current classification of cysteine protease clans and some of their families. With advances in protein structure and modeling, such as AlphaFold, better assignment of proteases to clans will be established. Much of the work done to understand the role and function of the sporulation-specific protease, YabG, has been performed in *B. subtilis*, with more recent efforts focused on characterizing the function of YabG in *C. difficile* (123–126, 128–132). Yamazawa et al. have shown that *B. subtilis* YabG possesses a catalytic dyad of His172 and Cys218 that allows for auto-processing and processing of substrates. Recently, it was shown that *C. difficile* YabG functions similarly, with a catalytic dyad of His161 and Cys207, and processes germination proteins CspBA and preproSleC along with regulating transcription of *cotA* and *cdeM* (128, 129). Based upon these data that identified the conserved catalytic dyad, we propose that YabG is an ideal candidate for clan CD in the MEROPS (Fig. 1).
